# Methods, models, mechanisms and metadata: Introducing the Nanotoxicology collection at F1000Research

**DOI:** 10.12688/f1000research.75113.1

**Published:** 2021-11-24

**Authors:** Iseult Lynch, Penny Nymark, Philip Doganis, Mary Gulumian, Tae-Hyun Yoon, Diego S.T. Martinez, Antreas Afantitis

**Affiliations:** 1 School of Geography, Earth and Environmental Sciences, University of Birmingham, Birmingham, B15 2TT, UK; 2 Institute of Environmental Medicine, Karolinska Institutet, Nobels väg 13, Stockholm, 17 177, Sweden; 3 School of Chemical Engineering, National Technical University of Athens, Athens, 10682, Greece; 4 National Health Laboratory Services, 1 Modderfontein Rd, Sandringham, Johannesburg, 2192, South Africa; 5 Haematology and Molecular Medicine, University of the Witwatersrand, 1 Jan Smuts Ave, Johannesburg, 2000, South Africa; 6 Water Research Group, Unit for Environmental Sciences and Management Potchefstroom, North West University, Potchefstroom, South Africa; 7 Department of Chemistry, College of Natural Sciences, Hanyang University, Seoul, 04763, South Korea; 8 Institute of Next Generation Material Design, Hanyang University, Seoul, 04763, South Korea; 9 Brazilian Nanotechnology National Laboratory (LNNano), Brazilian Center for Research in Energy and Materials (CNPEM), Campinas,, Sao Paulo, CEP 13083-970, Brazil; 10 Nanoinformatics department, NovaMechanics Ltd, Nicosia, Cyprus

**Keywords:** Nanomaterials, nanosafety, exposure, toxicity, risk assessment, mode of action, toxicogenomics, bio-nano interface, nanoinformatics, FAIR data, standardisation, regulation, green and sustainable nanomaterials, safe-by-design, environmental fate and behaviour

## Abstract

Nanotoxicology is a relatively new field of research concerning the study and application of nanomaterials to evaluate the potential for harmful effects in parallel with the development of applications. Nanotoxicology as a field spans materials synthesis and characterisation, assessment of fate and behaviour, exposure science, toxicology / ecotoxicology, molecular biology and toxicogenomics, epidemiology, safe and sustainable by design approaches, and chemoinformatics and nanoinformatics, thus requiring scientists to work collaboratively, often outside their core expertise area. This interdisciplinarity can lead to challenges in terms of interpretation and reporting, and calls for a platform for sharing of best-practice in nanotoxicology research. The F1000Research Nanotoxicology collection, introduced via this editorial, will provide a place to share accumulated best practice, via original research reports including no-effects studies, protocols and methods papers, software reports and living systematic reviews, which can be updated as new knowledge emerges or as the domain of applicability of the method, model or software is expanded. This editorial introduces the Nanotoxicology Collection in *F1000Research*. The aim of the collection is to provide an open access platform for nanotoxicology researchers, to support an improved culture of data sharing and documentation of evolving protocols, biological and computational models, software tools and datasets, that can be applied and built upon to develop predictive models and move towards *in silico *nanotoxicology and nanoinformatics. Submissions will be assessed for fit to the collection and subjected to the F1000Research open peer review process.

## Introduction

A key challenge in science currently is the push for novelty and impact, which may be at the expense of reproducibility and repeatability in highly competitive areas, although recent literature suggests that the narrative of science in crisis is not supported by evidence ^1^. Using a qualitative analysis approach, Nelsen *et al.* identified that reproducibility discussions centred on the incentive structure of science, the transparency of methods and data, and the need to reform academic publishing, in addition to discussions focused on (quality/purity of) reagents, on statistical methods, and on the heterogeneity of the natural world ^2^. Supporting researchers in documenting the latter three aspects, and in enhancing the transparency of methods and data, are core missions of F1000Research, with its focus on open access publication, transparent and open peer-review, the versioning of papers as new data emerges, and the range of article types offered, including descriptions of ***case studies, datasets, genomes, methods, protocols** and **software tools,** as well as **opinion articles, reviews, systematic and living systematic reviews** and **original research articles.*** This documentation of the current state of science is vital as so much knowledge is implicit, passed from researcher to researcher within lab groups, and is often not documented or fully captured in the written protocols and method descriptions in publications. F1000Research offers a platform to document this information in a fully citable way!

In the nanotoxicology arena, as indeed in all branches of toxicology, the need for novelty as a route to publication has led to a clear bias in the published literature towards effects studies, with negative or no-effect studies being much harder to publish due to a perceived lack of novel insights or new modes of action ^3^. Thus, a key goal of the F1000Research Nanotoxicology collection is to provide a home for well-designed, well-performed and well-documented studies at realistic concentrations and exposure conditions where low or no-effects are observed, in order to re-balance the literature and support development of predictive models based on balanced datasets. Additionally, low dose studies will allow new insights into the repair and recovery mechanisms that organisms induce in response to exposures, which are overwhelmed at higher exposures, including elucidation of normal housekeeping gene and protein expression, versus induction of repair mechanism such as anti-oxidants, DNA repair pathways, cell cycle arrest and protein clearance mechanisms, which have not yet been studied in depth for nanomaterials ^4, 5^. Correlations between nanomaterials properties and biological impact, which are the foundations of toxicology and required for risk assessment, including modelling approaches are welcomed, as these also provide the basis for design of safer greener and more sustainable nanomaterials and products that are safe by design. Progress towards single cell-level evaluations of accumulation of nanomaterials and analysis of heterogeneity of responses is also an important emerging direction ^6^. Mixture toxicity studies with nanomaterials and co-pollutants, including understanding the role of molecular interactions with the acquired biomolecule corona ^7^, are very welcome also, as the nanotoxicology community is providing leadership in this emerging topic. 

## Back to basics: documenting the lessons learned and the weight of evidence

A common challenge in the toxicology and nanotoxicology arena is that acquired knowledge gets “lost” over time, and ends up being “re-discovered“ later – examples of this include the potential for interference from nanomaterials with colorimetric assays ^8^ and resulting in indirect toxicity due to binding of medium components (proteins ^9^, micronutrients ^10^ or released cytokines ^11^), which was very topical 15 years ago, but is rarely mentioned now despite still being an important issue, although it has recently been highlighted in the context of high-throughput screening of nanomaterials ^12^. Similarly, leaching of fluorescent labels from particles and the consequent risks of mis-quantification of nanomaterial uptake in organisms and cells was very topical a decade ago in nanosafety research ^13, 14^, and is only recently being rediscovered in the microplastics and nanoscale plastics field ^15^, along with renewed exploration of the impacts of preservatives in commercial nanoplastic particle dispersions ^16^. Indeed, nanotoxicology itself has learned a huge amount from the particle toxicology community; for example knowledge about protein coronas dating back to early asbestos-studies ^17, 18^ was rediscovered within the field of nanotoxicology ^19– 21^. Thus, a key goal of the F1000Research Nanotoxicology collection is to bring together this “community knowledge” in a single location to provide a set of key issues to consider for contiguous fields and for those newly entering the arena of materials safety assessment, be they legacy nanomaterials, micro or nanoplastics, or emerging advanced 2D materials and composite materials. Publication of ****protocols**** and standard operating procedures, guidance on best practice and checklists of reporting criteria are all examples of what we encourage submission of to build up this milestone collection.

## Standardised and non-standardised test organisms and classical versus mechanistic toxicity assessment

A recurring debate in toxicology and nanotoxicology has been the need for standardisation of materials, methods, end-points and organisms to allow comparability of results versus the potential limitations of only using standardised organisms in terms of missing impacts in other species or at ecosystem level and the lack of mechanistic insights that can be gained from standard apical end-point tests ^22, 23^. Many of the standardised organisms used in toxicity testing and the accompanying test methods were developed for soluble or non-particulate chemicals, and as such significant work has been done over the last two decades to evaluate the suitability of the existing tests for use with nanomaterials (applicable also to micro- and nano-scale plastic particles) ^24– 26^. In parallel, the growing understanding of chemical versus physical or particle effects from nanomaterials, and the push towards alternatives to *in vivo* testing through development and utilisation of *in vitro* models such as air-liquid lung models ^27, 28^, spheroid-type models and other 3D culture approaches ^29, 30^, as well as the need for high throughput approaches and mechanistic insights to support grouping of nanomaterials and establishment of Adverse Outcome Pathways linking a molecular initiating event to a series of key events and an eventual adverse outcome at organism, population or community levels ^31, 32^, are all driving a push towards development of new models and new methods to support implementation of the 3R principles (Replacement, Reduction, and Refinement). 

Development and validation of new methods and models is extremely time consuming, and although more flexible and slightly quicker validation approaches have been proposed to keep pace with the rapid technological development of new methods, validation is still essential for regulatory acceptance and adoption of Safe by Design and alternative methods and approaches ^33, 34^, Thus, to support the steps within the control of the research community, including the pre-validation of methods through round-robins or interlaboratory comparisons (ILCs), the F1000Research collection will also provide a home for publication of results of nanomaterials ILCs and through our ****methods papers**** for complete documentation of the new *in vitro* or *in vivo* models and the accompanying ****protocols**** for application of the method. A key benefit of the F1000Research versioning approach is that as additional nanomaterials are assessed using the new method or protocol, or as comments from the research community on the protocol or method are received, this data can be added to the publication including additional authors where relevant (and with agreement of the original authors), through publication of a revised version, thus also facilitating streamlined extension of the domain of applicability of the method/protocol. The Data Availability Statement can also be updated with any new data in revised versions of papers. A similar process is also envisaged for computational models and software tools as discussed below. For the existing standardised model organisms, much of the literature on their underpinning biology, which is needed to enable interpretation of toxicological and ecotoxicological effects and outcomes, was published prior to the discovery of nanomaterials, and as such in-depth reviews of key biological pathways and processes that might be affected, either chemically or physically, by nanomaterials are also welcome as part of a set of reference publications on nanotoxicology, focussing on specific organisms or groups of related organisms.

## FAIRification of datasets and documentation of models: the importance of agreed metadata

Nanoinformatics, while developing rapidly, is still a long way off the robustness of chemoinformatics approaches for small molecules, where huge easily accessible databases such as ChemBL and others are well-established and data downloads can be automated and harmonised to meet the needs of modellers easily ^35^. This is the ultimate goal for nanotoxicology data also to drive the nanoinformatics wave, and the F1000Research collection will support this through publication of ****software articles**** describing models, ****data**** papers to document the datasets underpinning original articles and nanoinformatics papers, and through the aforementioned versioning approach which will allow tools and models to be easily updated as their domains of applicability are extended. An emerging area of interest is also the building of predictive models for nanomaterials (eco)toxicology ^36, 37^, including application of deep learning approaches ^38^ and integration of individual models into linked predictive models for risk assessment. While not yet applied to nanomaterials, comparison of model performance and establishment of consensus models is welcomed, and development of community standards around nanotoxicology metadata ^39^ to describe experimental and computational data from nanotoxicology, including through development of nanotoxicology-specific FAIR (Findable, Accessible, Interoperable and Re-usable) tools ^40^ and metrics ^41^ and nanotoxicity-specific databases linked to purpose built databases for omics data, exposure data etc. are welcome. Considerations of how to increase the re-usability of computational models is also an emerging topic that the F1000Research Nanotoxicology collection will play a key role in driving forward. While it’s too early to call it FAIRification of computational models, case studies on specific models and how they can be progressed through regulatory (e.g., OECD, ECVAM) validation processes and the role of documentation (e.g., of the underpinning hypotheses and datasets via the Quantitative Structure-Activity relationship (QSAR) model report forms, and the accompanying QSAR Prediction report form (QPAR)) in driving validation of such predictive models are topics that the Nanotoxicology collection will explore. 

## Conclusion

The F1000Research Nanotoxicology Collection offers researchers the opportunity to describe and fully document our models (biological and computational), methods (assays and protocols), mechanisms of action of nanomaterials including repair mechanisms and no-effect studies, datasets, best practice checklists, reporting guidelines and more, with full open access, transparent peer review and versioning to allow updating and extension of domains of applicability as new data become available. In the spirit of interdisciplinarity, the Nanotoxicology Collection will be looking to collaborate and interact with existing F1000Research gateways, such as NC3Rs and Chemical Information Science, to accelerate research in these intersectional spaces. We look forward to receiving your nanotoxicology publications!
